# Unveiling the nexus between rural population aging, technical efficiency, and carbon emissions in Chinese agriculture

**DOI:** 10.1371/journal.pone.0300124

**Published:** 2024-06-06

**Authors:** Hongen Song, Changyi Jiang, Zhaoming Sun

**Affiliations:** School of Economics and Management (College of Cooperatives), Qingdao Agricultural University, Qingdao, Shandong; University of Agriculture Faisalabad, PAKISTAN

## Abstract

**Introduction:**

According to the seventh national population census in China, the proportion of people aged 65 and above in the population reached 13.5%. The aging trend is more pronounced in rural areas, indicating that China has entered an aging society. This article focuses on agricultural carbon emissions in the context of aging, studying the impact of rural population aging on agricultural carbon emissions.

**Study objectives:**

Under the background of deepening population aging, let us discuss how to maintain the green and sustainable development of agriculture in China.

**Methodology:**

Fixed effects and mediating effects models are used. Technical efficiency is used as a mediating variable to discuss the relationship between rural population ageing, technical efficiency and agricultural carbon emissions.

**Results:**

This paper adopts the classical carbon emission calculation theory of IPCC to measure agricultural carbon emissions from 2010 to 2019, and China’s plantation carbon emissions show an "inverted U-shaped" trend, reaching a high level in 2015 and then starting to decline. In addition, the fixed-effects benchmark regression found that the aging of the rural population promotes agricultural carbon emissions, and the technical efficiency of agriculture suppresses agricultural carbon emissions. Finally, the mediating effect model is applied to explore the relationship between the three. Using technical efficiency as the mediating variable, it is found that under the masking effect, rural population aging will weaken agricultural carbon emissions through technical efficiency, thus achieving the suppression of agricultural carbon emissions.

**Policy recommendation:**

The formulation and modification of agricultural carbon-reducing policy row policies should take full account of the broader context of rural population ageing; increase the interconnectedness and interaction between rural population ageing and agricultural production technology, and actively play a positive role in promoting the efficiency of agricultural technology as a result of rural population ageing; and, in accordance with the actual situation of agricultural research, appropriately increase the strength of financial support for agriculture to improve agricultural technology and promote low-carbon development in agriculture.

## 1. Introduction

Part-time farming refers to the prevalence of part-time farming in a region, and is often expressed as the proportion of part-time farmers to the total number of farmers in the region. Most farmers in rural China are part-time farmers, and few are full-time farmers. However, with the increase of age, many old farmers can not go out to work, and they can only rely on agricultural production to increase their income, so the degree of part-time farming of old farmers is relatively low, and the proportion of income from farming is high. Thus, what is mentioned in the process of agricultural production is how the elderly farmers engaged in the process of agricultural production is achieved? Therefore, it is necessary to study the agricultural production of this type of elderly farmers on agricultural carbon emissions.

One of the primary contributors to global greenhouse gas emissions is the agriculture sector [1, 2]. Currently, agriculture stands as the second largest emitter of global GHGs, with emissions escalating approximately 1% annually [3, 4]. Despite utilizing only 7% of the world’s land to sustain a fifth of its population, China’s agricultural advancements have had environmental repercussions. The excessive employment of chemical fertilizers, pesticides, and machinery has amplified agricultural pollutants, exerting significant ecological pressure. Such activities contribute to 16.5% of global human-induced emissions, with the predominant source being chemical application in the farming sector. China’s "14th Five-Year Plan," introduced in 2021, underscores the third pivotal "energy conservation and emission reduction initiative". The fifth clause explicitly states an aim for major crops to achieve a 43% utilization rate of chemical fertilizers and pesticides.

Given China’s circumstances, the imperatives are twofold: first, there’s a pressing need for emission reductions; second, the escalating aging trend among the rural populace and the shifting agricultural labor dynamics require immediate attention. By international benchmarks, a society with 10% of its population aged 60 and above, or 7% aged 65 and above, is considered aging. When the percentage of those aged 65 and above reaches 14%, the society is deeply aging, and at 20%, it’s a super-aged society. Between 2010 and 2019, the average percentage of the elderly in China’s rural areas stood at 11.3%, indicating a significant shift towards a deeply aging demographic. Achieving the fertilizer and pesticide utilization targets set out in the 14th Five-Year Plan hinges on the enhancement of agricultural technological efficiency. Amidst the dual challenges of carbon reduction and aging, the intensifying aging trend in rural areas necessitates a review of technical efficiency. How might alterations in technical efficiency influence carbon emissions within the farming sector? Addressing this connection is paramount in our current agricultural endeavors and aligns with China’s vision of achieving "modernization in harmony with nature." Current investigations on crop carbon emissions in China encompass the following dimensions:

1. Research on the association between rural population aging and carbon emissions primarily occurs at national and regional levels. Nationally, a positive correlation exists between rural population aging and carbon emissions, while certain provinces indicate a negative correlation [5]. Beyond its direct impact, rural population aging has been observed to intensify CO2 emissions when GDP per capita acts as an intermediary [6]. Examining energy consumption, the aging population shifts the energy preferences of elderly rural farmers towards fossil fuels, leading to elevated carbon emissions [7]. Conversely, some research suggests that population aging may curtail carbon emissions. Data indicates a decline in emissions as the rate of population aging ascends [8]. Furthermore, at the national scale, the aging populace in developing countries seems to restrain carbon emissions [9]. From an age structure perspective, diverse influences of the population’s age framework on carbon emissions emerge. Notably, an aging population serves as a primary catalyst in augmenting carbon emissions, with urbanization and per capita consumption exacerbating this effect [10]. The nexus between population aging and carbon emissions is theorized to follow an inverted U-shape. This pattern suggests that carbon emissions exhibit an inverse U-shaped relation with population aging, aligning with the Environmental Kuznets hypothesis [11]. The maturation of the population diminishes carbon emissions via shifts in labor supply, leading to decreased consumption, further underpinning the U-shaped association [12]. While numerous studies have delved into the linkage between population aging and carbon emissions, the majority emphasize societal and household consumption effects, primarily focusing on population aging’s direct impact on emissions. Exploring rural demographics and technological advancement, research highlights diminished labor productivity among middle-aged and elderly workers [13]. This decline hampers agricultural technological development [14] and constrains the elevation of technical efficiency. Additionally, elder farmers, often resistant to new methodologies, further limit the growth of agricultural technical proficiency [15]. Concerning the interrelation between crop carbon emissions and agricultural technical efficiency, existing literature establishes a strong correlation between the two [16]. Some argue technological advancements exacerbate carbon emissions [17], with provincial data revealing a direct link between agricultural technology and emissions [18]. Yet, on a broader national scale, the technological influence on emissions appears dual-phased: initially increasing, then diminishing over time [19]. This dual impact underscores the nonlinear influence of agricultural technical efficiency on carbon emissions [20]. By comparing rural population ageing between developed and developing countries, it was found that ageing is much higher in Europe, North America and Japan than in less developed countries in Asia, Africa and Latin America, which in turn affects rural development21 (Stloukal L, 2023) [21]. By examining the impact of rural population ageing on farmers’ cleaner production behaviour, it was found that rural population ageing inhibits farmers’ cleaner production behaviour through learning ability. On the contrary, rural population ageing promotes farmers’ cleaner production behaviour through factor substitution and behavioural imitation (Liu J, Du S, Fu Z, 2021) [22]. Tobias, in his study of greenhouse gases in Germany, found that the development of an ageing demographic structure increases carbon emissions through changes in the configuration of energy consumption. The aging of the rural population in Poland analyses the concept of the silver economy as a new model for the development of rural areas and the problems associated with it, and provides an opportunity for sustainable rural development (Krzyminiewska G, 2019) [23]. In the context of accelerated ageing of the rural population, global agricultural production is threatened by a number of factors, the most common of which are climate change and the prevalent problem of declining soil fertility, inferring that any factor affecting the rural population has an impact on agricultural production (Akdemir S, Miassi Y, 2020) [24].

In essence, while numerous studies emphasize agriculture, the majority incorporate population aging into their analytical models. Yet, a limited number investigate the influence of population aging on carbon emissions through specific impact pathways. In our study, we examine the repercussions of population aging on carbon emissions within a framework of "high-quality development," considering China’s unique challenges of pronounced rural population aging and prevalent smallholder farming. Utilizing provincial panel data from 2010 to 2019, we employ stochastic frontier analysis to determine technical efficiency. Building on this, we introduce a mediation model to discern the mechanism by which rural population aging affects carbon emissions in the crop sector. Specifically, we integrate agricultural technical efficiency into our analysis to ascertain whether it can serve as a mitigating factor against the adverse effects of rural population aging on crop industry carbon emissions.

## 2. Theoretical foundation

The aging rural population and smallholder production operations are a microcosm of Chinese agriculture. In rural areas, the majority of aging farmers are those with low levels of knowledge and traditional farming practices. Schultz’s application of human capital theory to agricultural production also identified for the first time the critical role of human capital for economic growth, especially in agriculture. Whereas in aging societies, young and middle-aged laborers move to cities, often these young and middle-aged are the ones with a better level of education than older farmers, leaving behind older farmers with lower levels of knowledge and less ability to learn, and the poor knowledge of farmers is one of the main reasons that prevent farmers from taking climate-friendly actions [25]. And according to Yang Jun’s conclusion [26]: the improvement of agricultural human capital helps to mitigate carbon emissions, while the aging of the rural population is detrimental to the improvement of agricultural human capital, so the aging of the rural population will not promote the development of the economic level of agriculture and will not mitigate carbon emissions from planting. Chinese society is vernacular [27], and Chinese farmers are deeply attached to the land and have a unique complex. For elderly farmers, land is a partner that has been with them for most of their lives, which contains both economic and spiritual values, and this differential order pattern of blood and geography established with villagers is the reason why farmers are reluctant to give up one a unit of area of land. At the same time, the elderly farmers are more sensitive to the possible risks associated with land transfer than the average farmer. The fact that land cannot be transferred restricts the development of large-scale agriculture, but often agricultural production with scale can reduce the input of agricultural production materials such as chemical fertilizers and pesticides, and since most of the elderly farmers are individual households who are unwilling to transfer their land, this will also promote agricultural carbon emissions to a certain extent. Hypothesis H1: The effect of aging rural population on carbon emissions from farming is positive.

In a backdrop of an aging rural populace, there’s an evident uptick in the deployment of agricultural technologies. The crux of the matter lies in enhancing the efficiency of these technologies to subsequently pare down carbon emissions. The most conspicuous change precipitated by rural aging is the labor migration. Amid China’s rapid urbanization and industrialization, the younger, able-bodied rural populace gravitates towards urban centers, exacerbating rural aging. Older farmers tend to overcompensate for diminished labor by ramping up the use of fertilizers and pesticides, aiming to bolster crop yield. Such practices not only lead to the overconsumption of agricultural resources but also contribute significantly to environmental degradation. This intensifies carbon emissions in the agriculture sector and fails to optimize the use of agricultural inputs.

The "agricultural labor supply effect" highlights the declining physical capabilities of an aging workforce, which invariably leads to a reduction in effective labor available for agricultural production. With the younger demographic migrating to urban areas, elderly farmers left behind witness a continual aging trend, further dwindling the available active workforce in farming. This demographic shift pushes older farmers towards labor-efficient agricultural methodologies, favoring crops that are conducive to mechanization and simpler to cultivate. Consequently, advancements in agricultural technology cater to the needs of this aging farming demographic, pushing technological innovations in a direction that suits their capabilities. A pronounced aging trend accentuates labor productivity, and as per economic and technological growth principles, technological advancements typically parallel increases in labor productivity [13]. As a result, older farmers adapt their agricultural practices, gravitating towards increased usage of farming machinery, larger-scale land management, or opting for specialized agricultural services. The most prevalent adaptation involves the embrace of innovative agricultural production techniques. Consequently, an aging rural demographic spurs growth in agricultural technical efficiency and propels technological advancements in farming.

Agricultural technical efficiency delineates the proportion of actual agricultural output to the potential maximum yield attainable with existing technology. It serves as an index to gauge the efficacy of producers in harnessing available technologies, with efficiency values oscillating between 0 and 1 [28]. As such, with an enhancement in agricultural technical efficiency, there’s a subsequent surge in the optimization of agricultural resources. This heightened efficiency extends to critical agricultural inputs such as fertilizers, pesticides, and plastic mulches, which collectively acts as a deterrent to escalated carbon emissions from farming.

**Fig 1:**
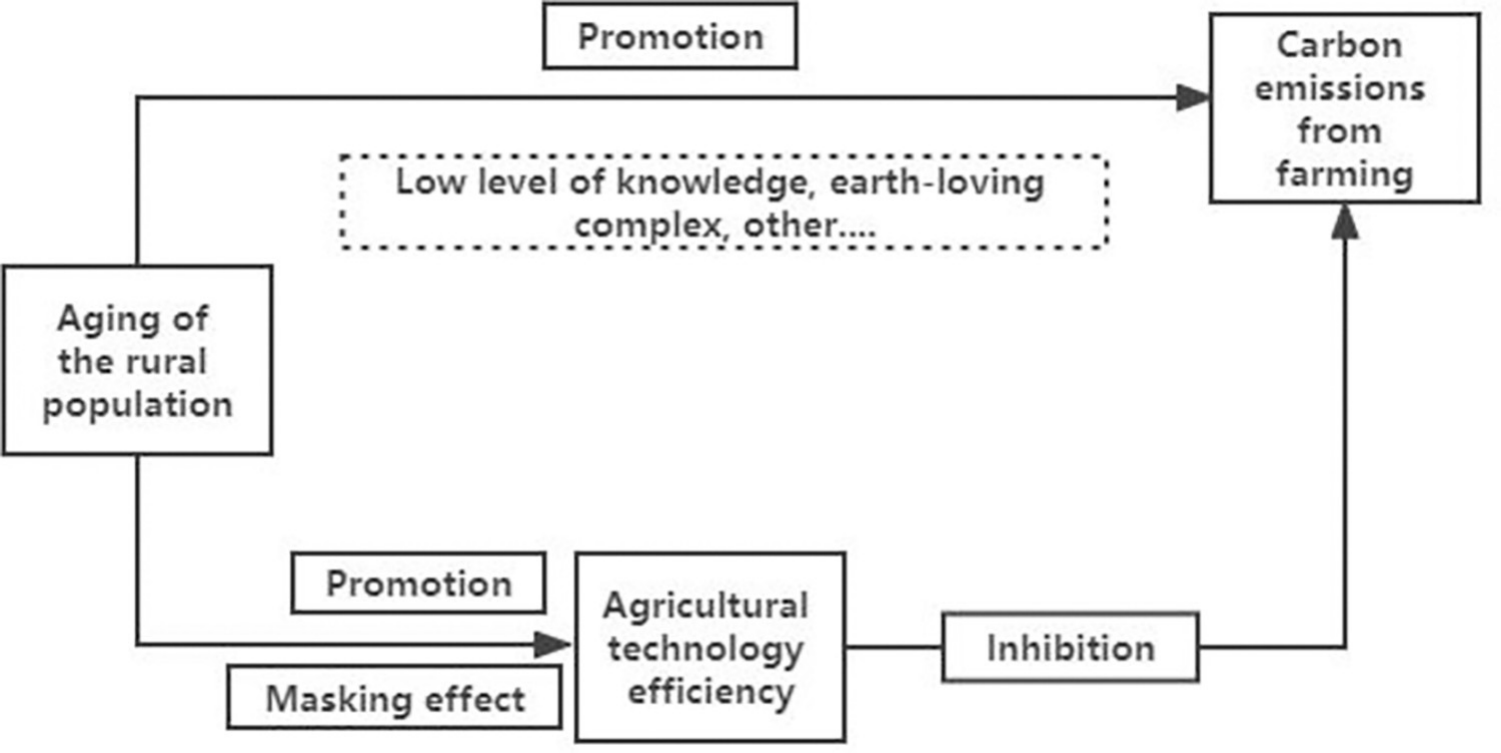
Specific theoretical analysis framework of the impact of rural population aging on agricultural carbon emissions.

Therefore, a frame diagram of the relationship between the three is made, as shown in Fig 1.

Hypothesis H2: Agricultural technical efficiency acts as an intermediary in the influence of an aging rural populace on carbon emissions from farming. Through bolstered agricultural technical efficiency, an aging rural demographic can ameliorate carbon emissions from cultivation.

## 3. Materials and methods

Reasons for model selection: The Agricultural Carbon Emission Estimation Model (Eq 1) was chosen to calculate agricultural carbon emissions and obtain relevant values. To study the relationship between rural population aging, technological efficiency, and agricultural carbon emissions, it is necessary to first use the fixed effects model (Eq 2) to directly examine the relationship between rural population aging and agricultural carbon emissions. Secondly, the mediating effects model (Eq 3) is used to assess the relationship between rural population aging and agricultural technological efficiency, considering the inclusion of the mediating variable of agricultural technological efficiency. Therefore, the fixed effects model and the mediating effects model were chosen.

### 3.1 Model setting for carbon emission measurement of plantation industry

In this study, the principal contributors to carbon emissions in cultivation—fertilizer, pesticide, agricultural film, diesel, tillage, and irrigation—are highlighted, aligning with the sources extensively recognized by researchers [19, 29, 30]. Utilizing the carbon accounting methodology proposed by the United Nations Intergovernmental Panel on Climate Change (IPCC), a carbon emission model for the plantation sector is established.


$$E=\Sigma E_{i}=\Sigma (C_{i}e_{i})\underset{x\to \infty }{\lim}$$ (1)

E, is the total amount of carbon emissions from cultivation; Ei, is the carbon emissions generated by the ith cultivation carbon source; Ci, is the amount of carbon sources from cultivation; ei, is the carbon emission coefficient (see Table 1).

**Table 1:** Carbon sources, carbon emission factors and sources in the plantation industry.

Carbon Source	Emission factor	Data reference source
Nitrogen fertilizer	2.116 t C /t	Chen Shun et al., 2015 [31]
Phosphate fertilizer	0.636 t C /t	Chen Shun et al., 2015 [31]
Potash	0.180 t C /t	Chen Shun et al., 2015 [31]
Compound fertilizer	0.977 t C /t	Zhang Guangsheng et al., 2014 [32]
Pesticides	1.793kg C /kg	Liu Xunhao et al., 2014 [32]
Agricultural film	5.18kg C /kg	(Chenget al., 2011) [33]
Diesel	0.862kg C /kg	IPCC(2006); China Energy Statistics Yearbook
plowing	312.60kg C /km2	(Fenlin Ng et al., 2007) [34]
Irrigation	266.48 kg C /hm^2^	West等, 2002 [35]

Note: C:CO2 = 1:3.67 was used for the conversion coefficient of C to CO2. compound fertilizer was borrowed from Zhang Guangsheng and Wang Shanshan (2014) [29], and the arithmetic mean was taken for nitrogen, phosphorus and potassium fertilizers. The diesel carbon emission factor was calculated using the carbon content of diesel given by IPCC (2006) and referring to the diesel fuel conversion factor and calorific value from the China Energy Statistical Yearbook.

### 3.2 Benchmark model setting

The following basic econometric model was set up to examine the impact of the aging rural population on carbon emissions from plantations:

$$CE_{it}=\alpha_{0}+\alpha_{1}Old_{it}+\alpha_{2}Control_{it}+\mu_{i}+\varepsilon_{it}$$ (2)

In the model, denotes each province and city (district), t, denotes the time (year), is the parameter to be deem, CEit, is the logarithm of carbon emissions from plantation, Oldit, is the aging of rural population, Control, is the control variable, *μ*_*i*_ is the province fixed effect, and *ε*_*it*_ is the random disturbance term.

### 3.3 Agricultural technology efficiency measurement

To gauge agricultural technical efficiency, two prevailing methodologies are employed: the Stochastic Frontier Analysis (SFA) and the Data Envelopment Approach (DEA). Given that the SFA integrates both the production function and random variables, aligning more congruently with agricultural production’s intrinsic dynamics, this study opts for the SFA. To ensure the model mirrors actual production conditions and augments both adaptability and comprehensiveness, the transcendental logarithmic production function is incorporated [36–38]. The model is delineated as follows:

$$\begin{array}{l} lnY_{it}=a_{it}+\beta_{Ait}\ln M_{it}+\beta_{kit}lnK_{it}+\beta_{Lit}lnL_{it}+\beta_{Dit}\ln N_{it}+\beta_{t}T+\beta_{AKit}\ln M_{it}lnK_{it}+\\ \beta_{ALit}\ln M_{it}lnL_{it}+\beta_{ADit}\ln M_{it}\ln N_{it}+\beta_{KLit}lnK_{it}lnL_{it}+\beta_{KDit}lnK_{it}\ln N_{it}+\beta_{LDit}lnL_{it}\ln N_{it}+\\ 0.5\beta_{AAit}{\left(\ln M_{it}\right)}^{2}+0.5\beta_{KKit}{\left(lnK_{it}\right)}^{2}+0.5\beta_{LLit}{\left(lnL_{it}\right)}^{2}+0.5\beta_{DDit}{\left(\ln N_{it}\right)}^{2}+0.5\beta_{tt}T^{2}+\\ \beta_{ATit}\ln M_{it}\times T+\beta_{kTit}lnK_{it}\times T+\beta_{LTit}lnL_{it}\times T+\beta_{DTit}\ln N_{it}\times T+\upsilon_{it}-\mu_{it}\\ Y_{it}=\alpha_{0}+\alpha_{1}LnX_{it}+\partial_{2}Control_{it}+\mu_{it}+\varepsilon_{it} \end{array}$$ (3)

*Y*_*it*_ which refers to the agricultural output of each province, using the total output value of agriculture, forestry, animal husbandry and fishery in each province., *M*_*it*_, *K*_*it*_, *L*_*it*_ and *N*_*it*_ denote land input, capital input, labor input and machinery input, respectively. Land input is the total cropping area of each province; capital input is agricultural material input, using the discounted amount of agricultural fertilizer application in each province; labor input is the number of agricultural laborers, using the primary industry employees; machinery input is the total power of agricultural machinery in each province. *T*, denotes the time, a_*it*_, denotes the constant term, *β*_*it*_ is the parameter to be estimated for the primary, interaction and squared terms of land input, capital input, labor input and machinery input, is the random error term, and *μ*_*it*_ is the agricultural efficiency loss term of the ith province. Assume that *μ*_*it*_ is independent of *υ*_*it*_ and follows a non-negative half-normal distribution with a mean of *Y*_*it*_^*U*^ and a variance of *σ*_*μ*_^2^.

### 3.4 Intermediary effects model

In this section, a three-step regression method [39] by Zhonglin Wen was used to test for mediating effects, with the following equations:

$$CE_{it}=\partial_{0}+\partial_{1}Old_{it}+\delta Y_{it}+e_{it}$$ (4)

$$TE_{it}=\beta_{0}+\beta_{1}Old_{it}+\delta Y_{it}+e_{it}$$ (5)

$$CE_{it}=c_{0}+c_{1}Old_{it}+c_{2}TE_{it}+\delta Y_{it}+e_{it}$$ (6)

*Old*_*it*_ is the degree of aging of the rural population, *CE*_*it*_ is the intensity of carbon emissions from cropping is the core explanatory variable of interest in this paper; *TE*_*it*_ is the technical efficiency of agriculture, which is the mediating variable; *Y*_*it*_ is the control variable; ∂, *β*, *c*, and *δ* are the parameters to be estimated; and *e*_*it*_ is the random error term. ∂_1_ is the total effect of the degree of aging of the rural population affecting the technical efficiency of agriculture, *β*_1_*c*_2_ is the indirect effect, and *c*_1_ is the direct effect, that is: ∂_1_ = *c*_1_+*β*_1_*c*_2_。

### 3.5 Variable selection and data sources

The explanatory variable in this paper is carbon emissions from the cropping sector, calculated using Eq (2) according to the IPCC recommendations. The core explanatory variable is agricultural technical efficiency, which is analyzed by stochastic frontier analysis, while the transcendental log production function is selected in order to be close to the actual production situation and enhance the flexibility as well as inclusiveness of the model [36–38]. In this paper, technical efficiency is defined as the maximisation of agricultural output for a given set of agricultural production inputs. Inputs are selected as land inputs, capital inputs, labour inputs and machinery inputs, and outputs are the total output value of agriculture, forestry, animal husbandry and fisheries in each province. The land input is the total crop area in each province; the capital input is the input of agricultural materials, using the pure amount of agricultural fertilizer applied in each province; the labour input is the number of agricultural workers, using the number of employees in the primary industry; and the machinery input is the total power of agricultural machinery in each province.

Control variables: Control Variables: Multiple determinants influence the carbon emissions within the cultivation sector. Drawing insights from extant literature, notably Jinyu Han et al., 2021 and Junqi Ma et al., 2021 [40, 41], this study identifies several control variables, detailed in Table 2.(1)Agricultural Infrastructure: This is gauged by the ratio of the effective irrigated region to the entire sown crop area. Contemporary rural infrastructure is heavily contingent upon carbon-intensive materials like steel and cement, as well as fossil fuels like diesel. Mismanagement in this regard jeopardizes the "double carbon" objective. (2) Food Crop Operation Scale: Quantified by the sown area of food crops (in million hectares). A more expansive operation inherently follows the law of diminishing returns. As a result, incremental inputs in agricultural practices wane, leading to a concomitant reduction in the carbon footprint of the cultivation sector.(3) Natural Disaster Rate: Defined as the ratio of the area impacted by disasters to the total sown crop area. Agriculture is inherently susceptible to natural calamities, underscoring the importance of considering extreme scenarios in production processes. (4) Agricultural Financial Support: Quantified as the local general public budgetary expenditure on agriculture, forestry, and water management relative to the total crop sown area (billion yuan/thousand hectares). Such financial backing ensures stability in agricultural endeavors. As farm subsidies grow, so does the incentive for farmers to produce. Concurrently, enhanced subsidies encourage farmers to adopt greener methods, incorporating elements like organic fertilizers, which subsequently diminish carbon emissions in the cultivation sector. (5) Rural Population Aging: Determined by the percentage of the rural populace aged 65 and above in the overall rural community. This factor, previously discussed, is believed to augment carbon emissions in agriculture. (6) Agricultural Labor Force: Calculated using the formula: (total agricultural output/combined output of agriculture, forestry, livestock, and fisheries) * cumulative employment in these sectors. The potential shift in this labor force can influence carbon emissions from farming.

**Table 2:** Variable definitions and descriptive statistics.

Variable name		Unit	Average value	Standard deviation	MIN	MAX	Selection of Variables
Carbon emissions from plantations	Calculated through carbon emission factors (tonnes)	ton (loanword)	408.231	291.632	15.797	1 230.068	Calculated using Eq 1
Technical efficiency in agriculture	Calculated using the SFA model	-	0.521	0.222	0.162	0.989	Calculated using Eq 3
Intensity of application of agrochemicals	Fertiliser application/area sown to crops (tonnes/million ha)	Tonnes/hectare	0.374	0.141	0.112	0.800	Reference 39
Agricultural infrastructure	Effective irrigated area/total sown area of crops (thousands of hectares)	%	0.456	0.205	0.172	1.234	Reference 39
Scale of food crop operations	Area sown with food crops (10,000 ha)	hectares	374.739	321.055	4.652	1433.810	Reference 39
Natural disaster rate	Area affected by crops/area sown with crops (thousands of hectares)	%	0.162	0.123	0.000	0.696	Reference 39
Financial support for agriculture	Expenditure on agriculture, forestry and water affairs"/total sown area of crops in local general public budget expenditure (billion yuan/thousand hectares)	Yuan/ha	0.257	0.627	0.025	6.602	Reference 40
Ageing of the rural population	Ratio of rural population aged 65+ to total rural population	%	0.113	0.034	0.050	0.229	Reference 40
Plantation labour force	(Gross value of agricultural output/gross value of agricultural, forestry, livestock and fisheries output)*total employment in agriculture, forestry, livestock and fisheries	classifier for individual things or people	0.100	0.053	0.003	0.3	Reference 40

To mitigate the influence of the recent pneumonia outbreak on agricultural outputs, the research confines its scope to the years 2010–2019. The study draws on panel data from 31 provinces, inclusive of autonomous regions and centrally-administered municipalities. Data pertinent to carbon emissions in the cultivation sector, agricultural technical efficiency, agricultural infrastructure, scale of food crop operations, disaster rates, financial backing for agriculture, and farming labor force statistics have been sourced from the China Statistical Yearbook. Information concerning the aging rural demographic is credited to the China Population and Employment Yearbook.

## 4. Empirical results and analysis

### 4.1 Analysis of the current status of carbon emissions from plantations, and the ageing of the rural population

**Fig 2:**
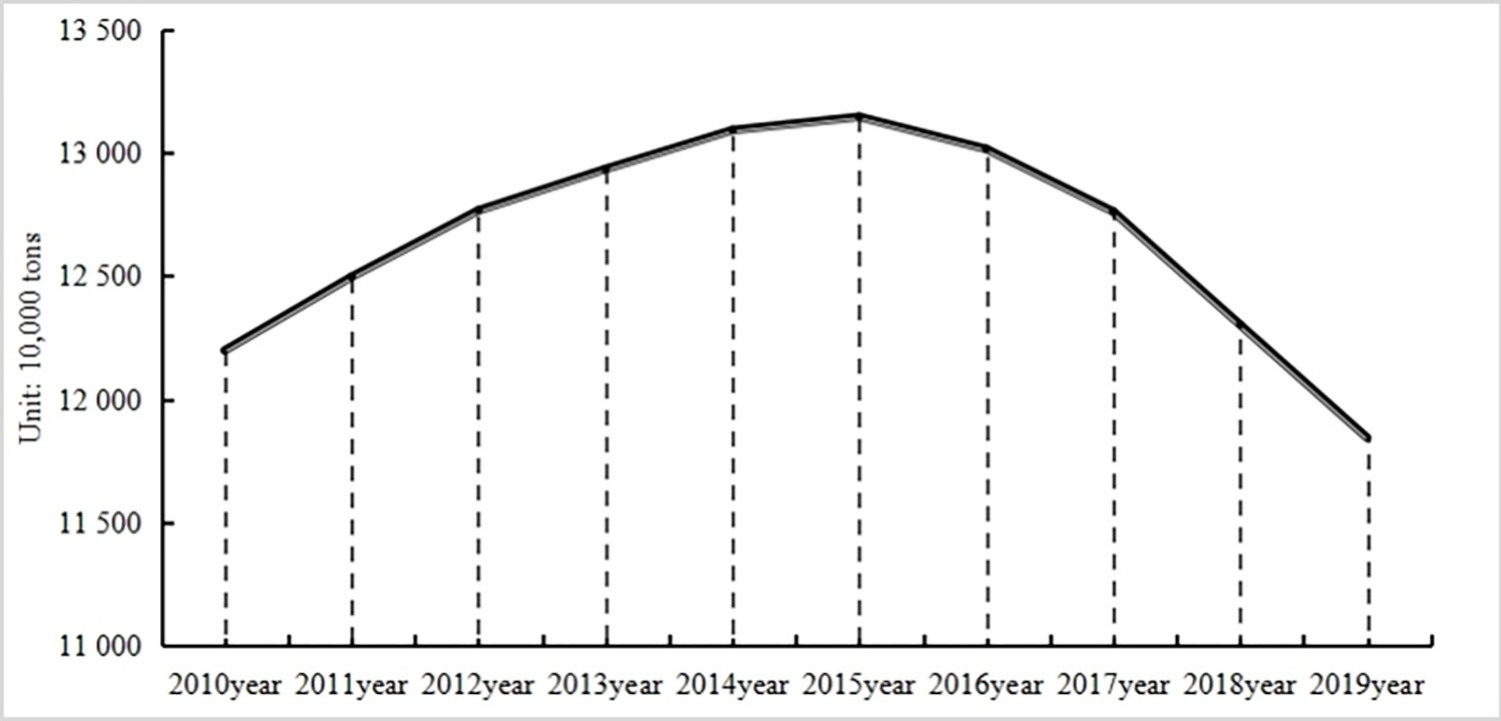
Changes in carbon emissions from cultivation in 31 provinces and regions nationwide, 2010–2019.

Over the decade from 2010 to 2019, China’s carbon emissions from the cultivation sector exhibited an "inverted U-shaped" trajectory (refer to Fig 2). Carbon emissions peaked in 2015 at 131,447,700 tons. Subsequently, there was a decline, with emissions dropping to 118,439,200 tons in 2019—a decrease of 9.90% from 2015 and 2.90% from 2010.

In early 2015, the then Ministry of Agriculture introduced an action plan targeting the reduction of chemical fertilizer and pesticide usage. The plan outlined a goal to attain zero growth in the utilization of these chemicals by 2020, emphasizing reliance on scientific management to optimize their use, fostering sustainable development. Consequently, post-2015 saw a dip in carbon emissions from the cultivation sector, predominantly attributed to the diminished use of pesticides and fertilizers—both major contributors to carbon emissions. In 2019 alone, emissions stemming from these two sources totaled 70,476,300 tons, comprising 59.50% of the cultivation sector’s carbon emissions. Thus, enhancing the efficiency of pesticide and fertilizer application is paramount for reducing the cultivation industry’s carbon footprint.

**Fig 3:**
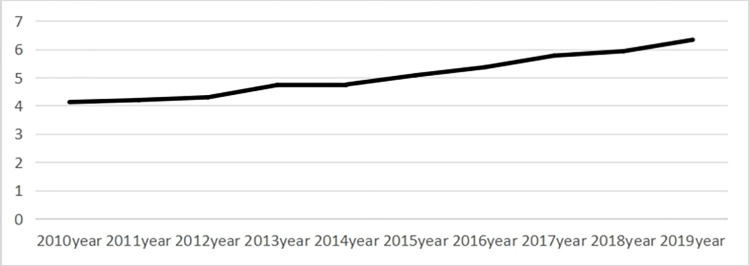
Changes in the ageing of the rural population in 31 provinces and districts nationwide, 2010–2019.

The ageing of the rural population shows an upward trend. As shown in (refer to Fig 3, rural population ageing shows a straight upward trend during the period 2010–2019. China’s two-child policy was liberalised in 2013, and the birth rate began to rise, but the proportion of older people is still large. 2021 saw the opening of the three-child policy, with a fertility rate of 7.52%, and 2022 saw a fertility rate of 6.77%. We can see that with the relaxation of the policy, the fertility rate declines rather than rises, and this very low fertility rate results in the aging phenomenon at the bottom coming to the fore. Overall, aging is increasing in both urban and rural areas, driven by low birth rates caused by family planning policies. As health care continues to improve, life expectancy has risen considerably and mortality rates have fallen, leading to an increasing number of older people in the country, while negative fertility growth has reduced population growth, resulting in an ageing population structure in both urban and rural areas. This has resulted in a linear upward trend.

### 4.2 Analysis of baseline regression results

Eq 1 was employed for the analysis. The outcomes of the baseline regression are presented in Table 3. Columns (1) and (2) delineate the findings from the fixed-effects and mixed-effects models, respectively. Column (3) conducts further assessment using random effects. It is evident from the table that all three methods yield significant baseline regression results. However, the coefficient related to the aging of the rural population exhibits the highest significance in the fixed-effects model. Thus, the fixed-effects model is selected.

**Table 3:** Baseline regression results.

	Fixed effects	Mixing effect	Random effects
	(1)	(2)	(3)
Aging of the rural population	4.1655***	3.5519***	3.5519***
	(5.1375)	(4.6660)	(4.6931)
Agricultural Infrastructure	0.8622***	0.8498***	0.8498***
	(5.8919)	(4.1791)	(5.8378)
Agricultural financial support	-0.0890*	-0.1053**	-0.1053**
	(-1.8523)	(-2.1670)	(-2.2247)
Plantation labor	-0.0425	-0.0419	-0.0419
	(-1.5251)	(-1.1384)	(-1.5105)
Natural disaster rate	0.0685	0.1549	0.1549
	(0.3300)	(0.6681)	(0.7779)
Food crop operation scale	0.8390***	0.8368***	0.8368***
	(29.9774)	(23.0242)	(30.0465)
intercept distance	-1.4430***	-1.3637***	-1.3637***
	(-5.8641)	(-5.1818)	(-5.6267)
N	310	310	310
R2	0.8709	0.8721	

Note: Standard errors are represented in parentheses, and *, **, and *** indicate statistical significance at the 10%, 5%, and 1% significance levels, respectively.

### 4.3 Agricultural technical efficiency measurement results and analysis

In this section, we utilize the STATA 16 software to conduct the maximum likelihood estimation of the transcendental log production function. Notably, the majority of the variables are significant at a 0.01 confidence level. The Wald test yields a value of 8267.53, with a p-value registering at 0.000, indicating a robust model fit. This supports the appropriateness of selecting the transcendental log production function model. *η* has an estimated value of 0.029, which significantly rejects " *H*_0_:*η* = 0 at the confidence level of 0.001 ", arguing that technical efficiency improves over time. *γ* has an estimated value of 0.997, calculated as " *γ* = *σ*_*U*_^2^/(*σ*_*V*_^2^+*σ*_*U*_^2^)", and the result is close to 1. Most of the composite disturbance term originates from the inefficiency component, justifying the use of the stochastic frontier model. Temporally, from 2010 to 2019, the agricultural technical efficiency of each province witnessed rapid improvement. Inefficiencies were largely addressed by 2016, with 14 provinces, including Beijing, Hebei, Liaoning, Jiangsu, Zhejiang, Fujian, Shandong, Henan, Hubei, Hunan, Guangdong, Guangxi, Hainan, and Sichuan, achieving high efficiency by 2019. There’s a distinct regional pattern in China’s agricultural technical efficiency, characterized by higher efficiency in the eastern provinces and lower efficiency in the west. The average agricultural technical efficiency across provinces stood at 0.559 in 2019, marking a 15.5% increase since 2010. This suggests that there remains potential for further optimization and progress.

### 4.4 Trend analysis of rural population aging, agricultural technical efficiency and carbon emissions from plantations

To elucidate the interplay among the three components, reference is made to the subsequent Table 4. Table 4 is based on the classification, which is the official Chinese standard for the basis of classification. Given that the carbon emission from the cultivation industry is an absolute figure, we use its intensity for a comparative analysis. It becomes evident that regions with pronounced aging of the rural population are primarily situated in the eastern and central parts, including areas like Shanghai, Zhejiang, Shandong, Hunan, and Hubei, with a few exceptions in the western and northeastern areas, such as Chongqing, Sichuan, and Gansu. Regions exhibiting commendable agricultural technical efficiency include Fujian, Guangdong, Hebei, Jiangsu, Shandong, and Zhejiang, which are predominantly located in the eastern and central regions. Meanwhile, provinces with a notably lower carbon emission intensity in the cultivation sector, such as Hebei, Shandong, Jiangsu, Henan, Hubei, and Guangxi, are majorly clustered in the eastern and western regions.

**Table 4:** Average values of rural population aging, agricultural technical efficiency, and carbon emission intensity of plantation by region, 2010–2019.

Region	Province	Carbon emission intensity of planting industry	Aging of the rural population	Agricultural technology efficiency
East	Beijing	0.28	0.11	0.58
	Fujian	0.18	0.12	0.88
	Hainan	0.21	0.09	0.62
	Guangdong	0.08	0.10	0.92
	Hebei	0.19	0.11	0.70
	Jiangsu	0.23	0.16	0.81
	Shandong	0.17	0.14	0.99
	Shanghai	0.24	0.15	0.41
	Tianjin	0.15	0.11	0.38
	Zhejiang	0.20	0.15	0.82
Middle	Anhui	0.20	0.13	0.45
	Hunan	0.28	0.13	0.56
	Hubei	0.21	0.13	0.64
	Henan	0.22	0.11	0.71
	Jiangxi	0.34	0.09	0.46
	Shanxi	0.35	0.10	0.31
West	Gansu	0.04	0.11	0.43
	Guangxi	1.86	0.11	0.59
	Guizhou	0.21	0.11	0.22
	Inner Mongolia	0.16	0.11	0.32
	Ningxia	0.22	0.07	0.23
	Qinghai	0.23	0.10	0.27
	Shaanxi	0.25	0.06	0.35
	Sichuan	0.01	0.15	0.64
	Xinjiang	0.16	0.08	0.20
	Yunnan	0.13	0.09	0.43
	Chongqing	0.24	0.18	0.34
	Tibet	0.25	0.06	0.44
Northeast	Heilongjiang	0.18	0.10	0.25
	Jilin	0.13	0.10	0.44
	Liaoning	0.18	0.13	0.79

Cross-referencing with Table 4, the average figures from 2010 to 2019 for rural population aging and agricultural technical efficiency in Shandong, Jiangsu, Zhejiang, and Hebei rank within the top 10, and they are all situated in the eastern part. Moreover, provinces with declining carbon emission intensities in the cultivation sector mirror this trend. Consequently, there are evident commonalities among the aging rural population, agricultural technical efficiency, and the carbon emission intensity of the cultivation sector, warranting deeper exploration into their relationships.

Over time, the aging of the rural population has been intensifying. As the elderly farmer demographic expands, there is an escalating demand for advanced agricultural mechanization and innovative technologies, thereby driving advancements in agricultural technical efficiency. A positive correlation emerges between the aging rural population and agricultural technical efficiency. On the other hand, the carbon emissions in the cultivation sector exhibit an inverted U-shaped trajectory, peaking in 2015 and subsequently declining. This shift can be attributed to the National Plan for Sustainable Agricultural Development, launched in China in 2015, which championed cost-effectiveness and efficiency. This policy initiative led to a significant reduction in the consumption of chemical fertilizers and pesticides. Enhanced agricultural technical efficiency bolstered the optimization of resources such as chemical fertilizers and pesticide films. As a result, as agricultural technical efficiency progressed, it played a pivotal role in curtailing carbon emissions in the cultivation sector.

### 4.5 Analysis of the mechanism of the impact of the aging of rural population on carbon emissions from plantations

#### 4.5.1 Analysis of model results

In this section, the mediating effects model is estimated, and the regression is performed using the three-step method of Chung-Lin Wen [42]. The main approach is to test the significance of ∂1, *β*1, and *c* 2 in the model sequentially. The significance of the total effect 1 is tested first, and then the significance of the product of coefficients is tested by testing *β*1 and *c*2. If ∂ 1, *β* 1 and *c*2 are significant and have the same sign, there is a mediating effect, and if it is different from *c*2 and significant, it is a masking effect. Based on regression findings, the aging of the rural population notably enhances carbon emissions in the plantation sector, validating hypothesis H1 at a 0.01 confidence level. However, upon integrating the mediating variable of agricultural technical efficiency, the coefficient of rural population aging becomes negative, significantly reducing carbon emissions in the plantation industry at a 0.1 confidence level. This integration indicates that as the rural population ages, it reduces carbon emissions in the plantation sector (Table 5), supporting hypothesis H2. Utilizing Mackinnon’s [43] method among others, it’s evident that the indirect effect of rural population aging on cropping sector carbon emissions serves as a masking effect rather than a mediating one. This masking effect is evident as the agricultural technical efficiency sign is positive in its indirect influence but contrasts the direct effect’s sign. The promotion of technical efficiency by an aging rural population results in diminished carbon emissions from the plantation industry. Considering the relationship between technical efficiency, rural population aging, and carbon emissions from crops, a "rebound effect" of carbon emissions might arise when the suppression rate of technical efficiency is outpaced by the growth rate of carbon emissions from crops. Furthermore, variations in technical efficiency, stemming from different stages of rural population aging, lead to diverse carbon emissions in the cropping sector. As technical efficiency acts as a catalyst, disparities among varied levels of rural population aging and carbon emissions from plantations magnify, forging a trade-off in carbon emission reduction. In situations of pronounced divergence, the influence of rural population aging on technical efficiency diminishes, converting a mediating effect to a masking one, potentially leading to a rebound. In essence, aging farmers indirectly influence technical efficiency in agricultural operations, which subsequently optimizes the use of fertilizers and pesticides, thereby curtailing carbon emissions from agriculture. The aging of the rural population is significant at the 0.01 confidence level (Table 5), and there is a large decrease in the coefficient estimates, and the absolute value of both effects (|∂1*β*1/*c*2|) is greater than the absolute value of the total effect, indicating that technical efficiency exerts a masking effect.

**Table 5:** Regression results of the masking effect model.

Explanatory variables	Carbon emissions from farming	Agricultural technology efficiency	Carbon emissions from farming
Aging of the rural population	3.3828***	2.8300***	-0.9773*
	(4.6019)	(8.2220)	(-1.7330)
Food crop operation scale	0.8385***	0.0204	0.8071***
	(30.1292)	(1.5633)	(41.6415)
Agricultural Infrastructure	0.8527***	0.4550***	0.1516
	(5.8397)	(6.6555)	(1.3982)
Agricultural financial support	-0.1032**	-0.0100	-0.0879***
	(-2.1802)	(-0.4499)	(-2.6742)
Natural disaster rate	0.1509	-0.0171	0.1772
	(0.7566)	(-0.1827)	(1.2806)
Plantation labor	-0.0414	0.0532***	-0.1234***
	(-1.4916)	(4.0914)	(-6.2354)
Agricultural technology efficiency			1.5407***
			(18.1030)
intercept distance	-1.3626***	-0.4588***	-0.6557***
	(-5.6047)	(-4.0304)	(-3.7881)
N	310	310	310
R^2^	0.8718	0.2942	0.9383

Note: Standard errors are represented in parentheses, and *, **, and *** indicate statistical significance at the 10%, 5%, and 1% significance levels, respectively.

#### 4.5.2 Robustness test

To bolster the robustness of our regression findings, we undertook endogeneity tests. Enhancing agricultural technical efficiency can foster better resource utilization. Concurrently, this efficiency boost can drive technological advancements, thereby reducing the carbon footprint of the plantation industry. Given the challenge of identifying an apt instrumental variable at the macro scale, we employed the lagged variable from a prior period of agricultural technical efficiency as a proxy, thereby addressing potential endogeneity concerns [37]. Table 6 displays the test outcomes. The regression indicates that the coefficients for both the lagged period of rural population aging and agricultural technical efficiency maintain their significance. Thus, our initial conclusions remain consistent.

**Table 6:** Endogeneity test results.

	Carbon emissions from farming	Technical efficiency in agriculture (lagged one period)	Carbon emissions from farming
Aging of the rural population	3.3828***	2.7730***	-1.1111*
	(4.6019)	(7.7532)	(-1.8862)
Food crop operation scale	0.8385***	0.0193	0.8039***
	(30.1292)	(1.3988)	(38.9477)
Agricultural Infrastructure	0.8527***	0.4617***	0.1534
	(5.8397)	(6.3722)	(1.3247)
Agricultural financial support	-0.1032**	-0.0108	-0.0897***
	(-2.1802)	(-0.4709)	(-2.6338)
Natural disaster rate	0.1509	-0.0272	0.1641
	(0.7566)	(-0.2724)	(1.1010)
Plantation labor	-0.0414	0.0543***	-0.1228***
	(-1.4916)	(3.9112)	(-5.7679)
Technical efficiency in agriculture (lagged one period)			1.5251***
			(16.8745)
intercept distance	-1.3626***	-0.4621***	-0.5944***
	(-5.6047)	(-3.8137)	(-3.2063)
N	310	279	279
R^2^	0.8718	0.2907	0.9371

Note: Standard errors are represented in parentheses, and *, **, and *** indicate statistical significance at the 10%, 5%, and 1% significance levels, respectively.

For further validation, we adjusted the regression model’s primary variables at the 2.5% level [44], trimmed the sample, and focused on data spanning 2011–2018 for regression. These specific results are detailed in column (4) of Table 7. When juxtaposed with the baseline regression, there’s minimal variance in regression coefficient values, sign orientation, or significance concerning carbon emissions in the cropping sector. This reinforces the masking relationship amongst rural population aging, agricultural technical efficiency, and carbon emissions in the cropping sector. The prevailing conclusions stand firm in this segment. Beyond its direct influence on enhancing carbon emissions from plantations, the aging rural populace curbs these emissions via improvements in agricultural technical efficiency.

**Table 7:** Results of the reduced-tail regression.

Explanatory variables	Carbon emissions from farming	Agricultural technology efficiency	Carbon emissions from farming
Aging of the rural population	3.5519***	2.9846***	-1.0610*
	(4.6930)	(8.4551)	(-1.8164)
Food crop operation scale	0.8498***	0.4531***	0.1494
	(5.8378)	(6.6744)	(1.3806)
Agricultural Infrastructure	-0.1053**	-0.0119	-0.0870***
	(-2.2247)	(-0.5369)	(-2.6457)
Agricultural financial support	0.1549	-0.0126	0.1745
	(0.7779)	(-0.1360)	(1.2618)
Natural disaster rate	0.8368***	0.0188	0.8077***
	(30.0465)	(1.4485)	(41.6323)
Plantation labor	-0.0419	0.0529***	-0.1237***
	(-1.5105)	(4.0915)	(-6.2538)
Agricultural technology efficiency			1.5456***
			(18.0744)
_cons	-1.3637***	-0.4611***	-0.6509***
	(-5.6267)	(-4.0796)	(-3.7668)
N	310	310	310
R^2^	0.8721	0.3015	0.9384

Note: Standard errors are represented in parentheses, and *, **, and *** indicate statistical significance at the 10%, 5%, and 1% significance levels, respectively.

## 5. Conclusions and recommendations

### 5.1 Main conclusions

In this study, we examined panel data from 31 provinces spanning 2010 to 2019, quantified carbon emissions from the plantation industry across these provinces, and dissected the impact mechanism of the aging rural populace on these emissions. Our findings include:

1. Over the decade, China’s carbon emissions in the plantation sector exhibited an "inverted U-shaped" trajectory. Emissions peaked at 131,447,700 tons in 2015 before receding annually, settling at 118,439,200 tons by 2019. Pesticide and fertilizer-related emissions, primary carbon contributors, reached 70,476,300 tons in 2019. Together, they represented 59.50% of the plantation sector’s carbon emissions. These emissions have consistently declined over the years, paralleling enhancements in agricultural chemical efficiency. Geographically, carbon emissions in the primary grain-producing regions surpassed the national average. Yet, these areas also witnessed steeper declines. By 2019, their emissions amounted to 76.958 million tons, a decrease of 10.16% from 2015 and 5.98% from 2010.
2. Regression analyses unveiled a direct correlation between the aging rural demographic and carbon emissions from plantations. As the aging trend intensifies, it appears to fuel higher emissions. As the elderly farmer population swells, they impact emissions through modifications in agricultural production—specifically in land utilization and chemical applications—aimed at boosting profitability.
3. The aging rural community influences the plantation sector’s carbon emissions by adjusting land use patterns and modulating pesticide and fertilizer applications, occasionally compromising environmental integrity in pursuit of enhanced yields.
4. The escalating aging trend within the rural populace fosters advancements in agricultural technical efficiency. This insight aligns with Jonas Pang’s [45] findings, suggesting that an aging rural workforce isn’t inherently detrimental. There’s a direct and positive linkage between rural aging and technical efficiency. Yet, when accounting for the mediating role of agricultural technical efficiency, the correlation with carbon emissions from the plantation sector inverts. By bolstering agricultural technical efficiency, the aging rural demographic curtails carbon emissions from plantations. Elevated technical efficiency enhances the effective use of carbon-intensive resources, like fertilizers and pesticides, leading to reduced agricultural chemical consumption and augmented crop yields. Most elderly farmers prioritize economic returns, often selecting innovative, high-efficiency, low-input, high-output agricultural technologies. This efficiency optimizes chemical use and boosts material efficiency in agricultural production, consequently dampening carbon emissions from the sector.
5. The aging rural community triggers a technical evolution within agricultural efficiency. Empirical data reveals that an aging rural demographic augments agricultural technical efficiency. As the number of elderly farmers grows, shifts in agricultural techniques and practices emerge. This, coupled with a dwindling efficient agricultural workforce, stimulates larger-scale farming, elevates technological standards, and propels advancements in agricultural technical efficiency. This not only minimizes material inputs but also refines resource use efficiency. The "14th Five-Year Plan to Promote Agricultural and Rural Modernization" emphasizes the necessity of technological leaps to ensure consistent, substantial yields, advocating for the adoption of contemporary tools and techniques to offset inherent water and soil resource deficits. The rising segment of elderly farmers undoubtedly catalyzes agricultural technological development, harmonizing with both sustainable agricultural aspirations and the dual mountains theory.

### 5.2 Discussion and limitations

Discussion: After the above arguments, we have concluded that rural population aging can be restrained through the improvement of agricultural technology efficiency to suppress agricultural carbon emissions. This contrasts with previous discussions among experts about the relationship between rural population aging and agricultural technology efficiency. For example, the result shows the opposite direction from the argument that elderly labor will lower marginal labor productivity level and restrain agricultural technology efficiency. In other words, deepening levels of aging are not entirely detrimental to sustainable agricultural development. In today’s era of continuous technological advancements, rural population aging can promote agricultural technological progress through inducement of technological changes. Consequently, by advancing agricultural technology, resource utilization efficiency can be improved, thus reducing agricultural carbon emissions to some extent. We should approach the issue of aging with a rational perspective.

#### Limitations

It is necessary to acknowledge the limitations of this work。Due to the unavailability of data, population data for rural areas in China, Hong Kong, Macao, and Taiwan is not accessible, and the sample size may be small. At the same time, due to a bias towards agricultural cultivation, there may be some deviations in the study of overall agricultural carbon emissions. However, overall, national publicly available population statistics are used, and the calculation method of agricultural carbon emissions has been recognized by scholars, so the experimental results are generally reliable and can be applied to further research. When conducting research on the aging of rural populations and agricultural carbon emissions, it is necessary to refer to past literature as the basis of the study. Reviewing literature can provide a theoretical foundation for the research topic. However, due to the different scope of the research topic, relevant literature may be limited. In this case, the lack of previous literature can be seen as an opportunity to fill the research gap and provide suggestions for future research directions in related fields.

### 5.3 Policy recommendations

1. It’s crucial to recognize the effects of the aging rural population on contemporary agricultural practices. When crafting or revising agricultural carbon reduction policies, it’s imperative to consider the overarching context of the aging rural demographic. This involves promoting a balanced age distribution within the agricultural labor force, leveraging the unique benefits of an aging rural populace, and thoughtfully guiding the farming strategies of elderly farmers, all while prioritizing food security.
2. There should be an enhanced synergy between the aging rural community and modern agricultural technologies. Embracing the positive impact of the elderly demographic in advancing agricultural efficiency is essential. Efforts must be directed towards bolstering the technical acumen of senior farmers, ensuring their proficient application of contemporary farming methods. Moreover, there’s a need to disseminate knowledge about ecological conservation and environmental protection. This will help elderly farmers grasp the significance of low-carbon farming in today’s China, prompting them to adopt carbon-conscious approaches throughout the agricultural value chain.
3. In light of current agricultural research findings, it’s advisable to bolster financial backing for advancing agricultural technologies, furthering the low-carbon trajectory of the plantation industry. Progressive agricultural methodologies will optimize resource utilization in farming, thus championing sustainable, carbon-efficient practices. Alongside this, a more robust framework for agricultural production services should be developed. By making these services more accessible to farmers and offering subsidies to the elderly participants, we can ensure senior farmers not only understand but actively engage with these services. Such an approach will invigorate their enthusiasm, enabling them to utilize these services, enhance technical efficiency, and subsequently minimize carbon footprints in the plantation sector.
